# Eicosapentaenoic Acid Rescues Cav1.2-L-Type Ca^2+^ Channel Decline Caused by Saturated Fatty Acids via Both Free Fatty Acid Receptor 4-Dependent and -Independent Pathways in Cardiomyocytes

**DOI:** 10.3390/ijms25147570

**Published:** 2024-07-10

**Authors:** Masaki Morishima, Pu Wang, Kosuke Horii, Kazuki Horikawa, Katsushige Ono

**Affiliations:** 1 Department of Food Science and Nutrition, Faculty of Agriculture, Kindai University, Nara 6318505, Japan; 2 Department of Applied Biological Chemistry, Graduate School of Agriculture, Kindai University, Nara 6318505, Japan; 2444670003v@nara.kindai.ac.jp; 3 Department of Pathophysiology, Oita University School of Medicine, Yufu 8795593, Japan; wangpucardio@163.com; 4 Department of Optical Imaging, Advanced Research Promotion Center, Tokushima University, Tokushima 7708503, Japan; horikawa.kazuki@tokushima-u.ac.jp; 5 Oita Shimogori Hospital, Oita 8700926, Japan

**Keywords:** eicosapentaenoic acid, oleic acid, palmitic acid, omega-3 polyunsaturated fatty acid, PUFA, FFAR4, CREB, Cav1.2, L-type Ca^2+^ channel

## Abstract

Dietary intake of omega-3 polyunsaturated fatty acids (eicosapentaenoic acid, EPA) exerts antiarrhythmic effects, although the mechanisms are poorly understood. Here, we investigated the possible beneficial actions of EPA on saturated fatty acid-induced changes in the L-type Ca^2+^ channel in cardiomyocytes. Cardiomyocytes were cultured with an oleic acid/palmitic acid mixture (OAPA) in the presence or absence of EPA. Beating rate reduction in cardiomyocytes caused by OAPA were reversed by EPA. EPA also retrieved a reduction in Cav1.2 L-type Ca^2+^ current, mRNA, and protein caused by OAPA. Immunocytochemical analysis revealed a distinct downregulation of the Cav1.2 channel caused by OAPA with a concomitant decrease in the phosphorylated component of a transcription factor adenosine-3′,5′-cyclic monophosphate (cAMP) response element binding protein (CREB) in the nucleus, which were rescued by EPA. A free fatty acid receptor 4 (FFAR4) agonist TUG-891 reversed expression of *Cav1.2* and *CREB* mRNA caused by OAPA, whereas an FFAR4 antagonist AH-7614 abolished the effects of EPA. Excessive reactive oxygen species (ROS) accumulation caused by OAPA decreased *Cav1.2* and *CREB* mRNA expressions, which was reversed by an ROS scavenger. Our data suggest that EPA rescues cellular Cav1.2-Ca^2+^ channel decline caused by OAPA lipotoxicity and oxidative stresses via both free fatty acid receptor 4-dependent and -independent pathways.

## 1. Introduction

A large number of epidemiologic studies demonstrate that higher saturated fat intake is associated with an increased risk of sudden cardiac death, suggesting that the effects of dietary saturated fat may be sufficient to cause heart diseases [1,2,3,4]. High levels of circulating saturated fatty acids are also associated with diabetes, obesity, and hyperlipidemia [5,6]. In the heart, the accumulation of saturated fatty acids has been proposed to play a role in the development of heart failure and arrhythmias [6,7,8]. The two major circulating fatty acids are saturated palmitic acid (C16:0) and monounsaturated oleic acid (C18:1) [9,10]. Palmitic acid is one of the most abundant fatty acids in human/animals and its overload induces lipotoxicity, resulting in apoptosis, endoplasmic reticulum stress, and reactive oxygen species production [11,12]. In addition, several recent studies have reported that plasma concentrations of palmitic acid were increased in a high-fat diet-induced obesity mouse model which has been used in a large number of publications to demonstrate lifestyle-related diseases in animals [7,8,9,10,13]. Exposure of high levels of palmitic acid to isolated cardiomyocytes is known to result in contractile dysfunction and apoptosis [7,14]. It is also recognized that palmitic acid-induced ROS production impairs cellular Ca^2+^ handling possibly through the decrease in L-type Ca^2+^ currents, increase in the open probability of SR Ca^2+^ release channels, slowing SR Ca^2+^ reuptake, and the activation of sarcolemmal Na^+^/Ca^2+^ exchange activity, ultimately leading to reduced SR Ca^2+^ content [7,15,16,17]. In contrast, numerous animal experiments and human epidemiological studies [18,19,20,21] have shown that omega-3 polyunsaturated fatty acids (PUFAs) exert beneficial effects on physical health. The strongest evidence for a valuable action of PUFAs has to do with cardiovascular diseases, causing a number of physiological changes such as decreasing heart rate and lowering blood pressure [22]. Interestingly, accumulating evidence from in vitro experiments has demonstrated that omega-3 PUFAs exert antiarrhythmic effects [23,24,25,26]. Eicosapentaenoic acid (EPA) has been shown to affect sodium channels [23] to protect cardiomyocytes against arrhythmias induced by high extracellular calcium, ouabain, isoproterenol, or lysophosphatidylcholine [24]. EPA and docosahexaenoic acid (DHA) are also known to regulate the activity of L-type Ca^2+^ channels, which plays an important role in reducing excessive excitability and increasing refractoriness of cardiac myocytes [25,26]. Thus, inhibition of Na^+^ and/or Ca^2+^ currents may account for the acute antiarrhythmic effects of omega-3 PUFA [23,24,25,26]. However, the underlying mechanisms associated with saturated fatty acid-induced long-term electrical changes of cardiomyocyte remain unclear [27,28,29].

Recently, the molecular targets for PUFAs were elucidated [30,31]. The free fatty acid receptor 4 (FFAR4) is a G protein-coupled receptor for endogenous medium- or long-chain fatty acids that attenuates metabolic diseases and inflammation. PUFAs are generally full agonists for FFAR4 [30,31] that are expressed in various cell types including cardiomyocytes [28,29]. It has been reported that high-fat diet-induced obesity and liver steatosis were more severe in FFAR4-deficient mouse than in wild-type mouse [32], suggesting the functional importance of this receptor in lipid pathology. Of note, Murphy et al. showed that FFAR4 in cardiac myocytes responds to endogenous fatty acids, reduces oxidative damage, and protects the heart from pathological stress. This could provide important translational implications for targeting FFAR4 in cardiovascular disease [33]. Furthermore, EPA was more effective than DHA at preventing lethal arrhythmias by inhibiting inflammasome and sympathetic innervation through activation of peroxisome proliferator-activated (PPAR) γ-mediated FFAR4-dependent and -independent signaling pathways after injury [34]. These results suggest that FFAR4 becomes an important site of action for EPA in the regulation of cardiac electrical activity. However, the role of EPA on cardiac electrical changes and transcriptional regulation of ion channels in the modification of these FFAR4-mediated signaling pathways has not been elucidated. Thus, the aim of this study was to investigate the possible beneficial effects of EPA and FFAR4 on saturated fatty acid-induced electrical changes in cardiac myocytes, focusing on the voltage-gated L-type Ca^2+^ channel.

## 2. Results

### 2.1. Actions of Saturated Fatty Acid on the L-Type Ca^2+^ Channel and the Cellular Excitability

**Figure 1:**
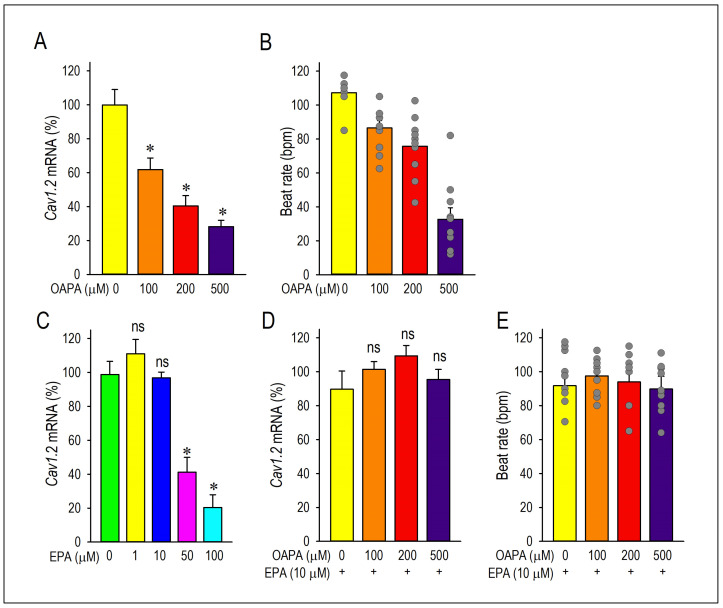
Long-term effects of fatty acid (OAPA) and EPA on the Ca^2+^ channel expression and cardiac automaticity. (**A**,**B**) Effects of OAPA on the expression of L-type Ca^2+^ channel isoforms (*Cav1.2*) mRNA and mean spontaneous beating rate (not normalized) of cardiomyocytes after application for 24 h. Cardiomyocytes were cultured with 100–500 μM palmitic/oleic acid (OAPA: 2:1) for 24 h. (**C**). Effects of EPA on the expression of *Cav1.2* mRNA. (**D**,**E**) Effects of 10 μM EPA on the expression of *Cav1.2* mRNA and mean spontaneous beating rate in combined with 100–500 μM OAPA. Data were normalized to *Cav1.2* mRNA expression in non-treated cardiomyocytes, which was designated as 100. Data are expressed as the means ± SE (n = 8). * *p* < 0.05 compared with the control group (EPA 0 μM or OAPA 0 μM). “ns” indicates statistical non-significance (*p* > 0.05) compared to EPA (-) or OAPA (-).

To determine whether cardiomyocyte excitability and automaticity can be affected by a high level of saturated fatty acid, we examined the effects of OAPA (100 to 500 μM) on neonatal mouse cardiomyocytes for 24 h. Since cardiomyocytes’ beating or automaticity was strongly influenced by the function of pacemaker ion channels, we first measured the mRNAs of these pacemaker ion channels in cardiomyocytes with OAPA by use of real-time PCR. In preliminary studies, OAPA significantly reduced the expression of *HCN4* and *KCNJ3* mRNAs. Interestingly, at the same time, the expression of *Cav1.2* L-type Ca^2+^ channel mRNAs were drastically reduced by OAPA. The expression of *Cav1.3* L-type Ca^2+^ channel mRNAs was also reduced by OAPA. Because the *Cav1.3* mRNA was expressed at much lower levels than that of *Cav1.2*, approximately ~3% of *Cav1.2* in neonatal cardiomyocytes, we only focused on the Cav1.2 L-type Ca^2+^ channel in this study. To investigate the possible effect of saturated fatty acid on cardiac automaticity in neonatal mouse cardiomyocytes, the spontaneous beating rate of cardiomyocyte was measured by live cell imaging system. As shown in Figure 1B, OAPA treatment significantly decreased the spontaneous beating rate in a dose-dependent manner. Interestingly, polyunsaturated fatty acid EPA also affected the expression of *Cav1.2* mRNA (Figure 1C). Although a 50 μM or higher concentration of EPA significantly decreased *Cav1.2* mRNA expression, lower concentration of EPA (1 μM or 10 μM) had a negligible effect on *Cav1.2* expression (Figure 1C). Given these results, we conclude that OAPA, and EPA with very high concentration, reduce the beating rate of cardiomyocytes in a concentration-dependent manner concomitant with the reduction in *Cav1.2* L-type Ca^2+^ channel expression in cardiomyocytes.

### 2.2. EPA Rescued OAPA-Induced Reduction in L-Type Ca^2+^ Channel

**Figure 2:**
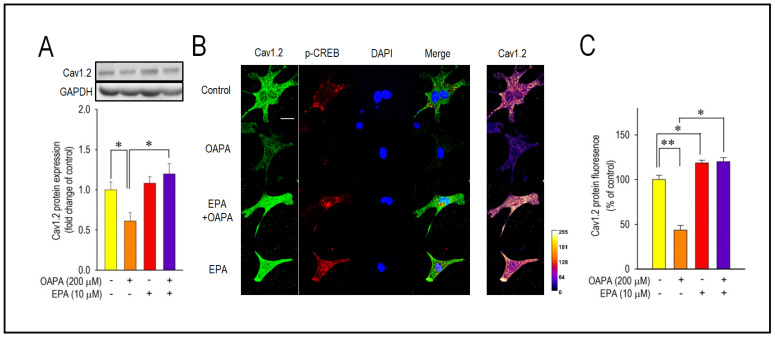
Effect of OAPA and EPA on the expression of Cav1.2 protein in neonatal mouse cardiomyocytes. (**A**) Representative Western blot and summary graph of Cav1.2 level after application with OAPA (200 μM) in the presence or absence of EPA (10 μM) for 24 h. (**B**) Expression and distribution of Cav1.2 and p-CREB as assessed by immunocytochemistry procedure. Cardiomyocytes were exposed to OAPA (200 μM) with or without EPA (10 μM) for 24 h. Cav1.2 for stained in green, p-CREB in red, and DAPI staining to visualize nuclei in blue. Scale bar = 20 μm. (**C**) The percentage of Cav1.2 fluorescence intensity was calculated. The signal intensity of Cav1.2 in non-treated myocytes (OAPA (-), EPA (-)) was set as 100%. Data are expressed as mean ± SE (n = 4). Asterisks indicate significant differences (* *p* < 0.05, ** *p* < 0.01).

**Figure 3:**
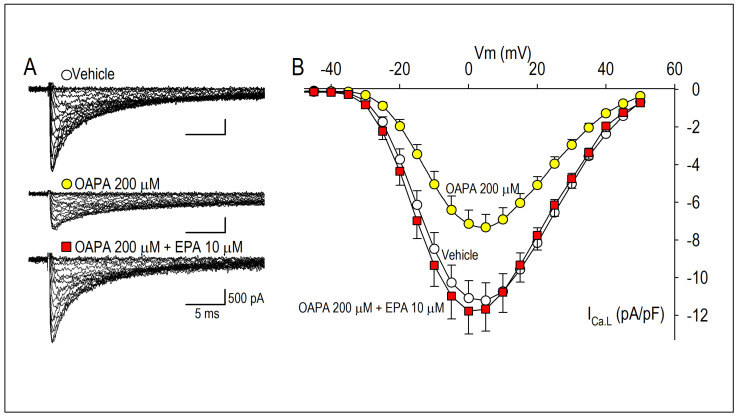
Effects of OAPA and EPA on *I*_Ca.L_ in rat neonatal cardiomyocytes. Cardiomyocytes were cultured with OAPA (200 μM) in the presence or absence of EPA (10 μM) for 24 h. Representative *I*_Ca.L_ traces in the vehicle and OAPA with or without EPA applied for 24 h (**A**), and their group data of current (I)–voltage (V) relationship (**B**). Current traces were obtained from a holding potential of −40 mV to test potentials up to 50 mV with 10 mV increments. Data are expressed as mean ± SD (n = 7).

The combined actions of OAPA and EPA on Cav1.2 expression and beating rate were then examined based on the results in Figure 1B,E. Interestingly, the reduction in *Cav1.2* mRNA expression caused by OAPA was all rescued by the presence of EPA (Figure 1A,D). Of note, even the highest concentration of OAPA (500 μM) was without effect on *Cav1.2* mRNA expression when 10 μM EPA was applied together (Figure 1D). These results suggest the rescue effect of EPA on the OAPA-mediated decline of the L-type Ca^2+^ channel when applied for 24 h. Consistent with these results, a decrease in the beating rate caused by OAPA was canceled in the presence of EPA (Figure 1E). In addition, a significant reduction in the protein expression levels of Cav1.2 caused by OAPA was completely rescued by EPA verified by Western blotting (Figure 2A), while EPA had an unchanged Cav1.2 protein level when OAPA was absent (*p* = 0.514). Furthermore, immunocytochemical analysis revealed that OAPA suppressed the expression of the Cav1.2 channel protein, which was completely rescued by EPA (Figure 2B,C). These results were all consistent with the changes in *Cav1.2* mRNA as shown in Figure 1. Functional modification of Cav1.2 was then confirmed by the electrophysiological method to explore the changes in the L-type Ca^2+^ channel current (*I*_Ca.L_). Since the saturated fatty acid OAPA strongly decreased the expression level of Cav1.2, which was blocked by EPA, a rescue of L-type Ca^2+^ channel current by EPA was expected. In conventional whole-cell patch clamp experiments using neonatal rat cardiomyocytes, *I*_Ca.L_ was recorded. Results in Figure 3 demonstrate that OAPA significantly reduced *I*_Ca.L_ when applied for 24 h, which was completely rescued by 10 μM EPA. Importantly, the current (I)–voltage (V) relationship was unchanged in the presence of OAPA and EPA, suggesting that the gating properties of the Cav1.2 channel was not modified by OAPA and/or EPA (Figure 3B).

**Figure 4:**
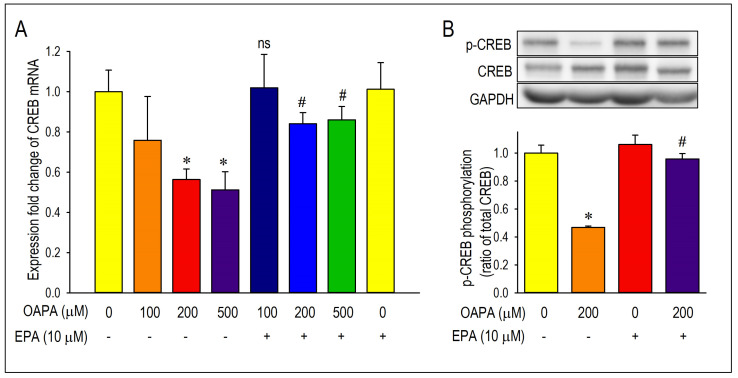
Long-term effect of OAPA and EPA on *CREB* mRNA expression and CREB phosphorylation in neonatal mouse cardiomyocyte. (**A**) Cardiomyocytes were cultured with 100–500 μM OAPA in the presence or absence of EPA (10 μM) for 24 h to assess the expression of *CREB* mRNA. (**B**) Changes in phosphorylated CREB (p-CREB) protein level in the nucleus of cardiomyocytes. Relative levels of proteins were determined by densitometry of the immunoblots. Data were normalized by taking the value of the control groups as 1.0. Data are expressed as mean ± SE (n = 5). * *p* < 0.05, vs. non-treated cardiomyocytes (OAPA (-), EPA (-)). # *p* < 0.05, vs. cardiomyocytes (OAPA (200 μM), EPA (-)) or (OAPA (500 μM), EPA (-)). “ns” indicates statistical non-significance vs. (EPA (-), OAPA (100 μM).

Activation of the transcription factor adenosine-3′,5′-cyclic monophosphate (cAMP) response element binding protein (CREB) as detected by phosphorylation of CREB has been recognized as an index of the initiation of Cav1.2 transcription [35]. Accordingly, the expression and the changes in *CREB* mRNA by OAPA were explored in the presence or absence of EPA. Importantly, *CREB* mRNA was reduced by OAPA in a dose-dependent manner, which was highly consistent with the changes in *Cav1.2* mRNA (Figure 1A) and Cav1.2 protein (Figure 2A). In accordance with the regulation of *Cav1.2* mRNA, EPA rescued the changes in *CREB* mRNA. A reduction in the phosphorylated component of CREB in the nucleus was also rescued by EPA, strongly suggesting that EPA rescues Cav1.2 protein through a transcriptional pathway involving CREB (Figure 4B).

### 2.3. Actions of FFAR4 in Cardiomyocytes

**Figure 5:**
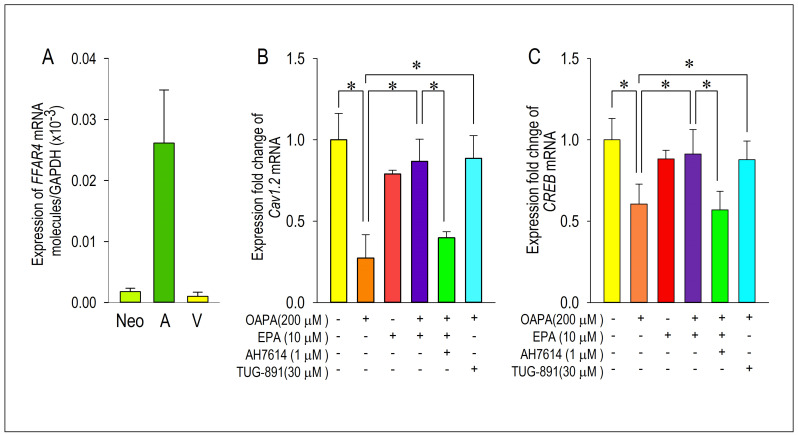
FFAR4 actions on Cav1.2 and CREB expression. (**A**) *FFAR4* mRNA expression levels in cardiomyocytes from neonatal mouse ventricle, adult mouse atrium, and adult mouse ventricle. (**B**,**C**) Cardiomyocytes were cultured with OAPA, EPA, a selective FFAR4 antagonist AH7614, and an FFAR4 agonist TUG-891 for 24 h. Data were normalized to *Cav1.2* mRNA expression in non-treated cardiomyocytes, which was designated as 100. Data are expressed as mean ± SE (n = 6). Asterisks indicate significant differences (* *p* < 0.05).

**Figure 6:**
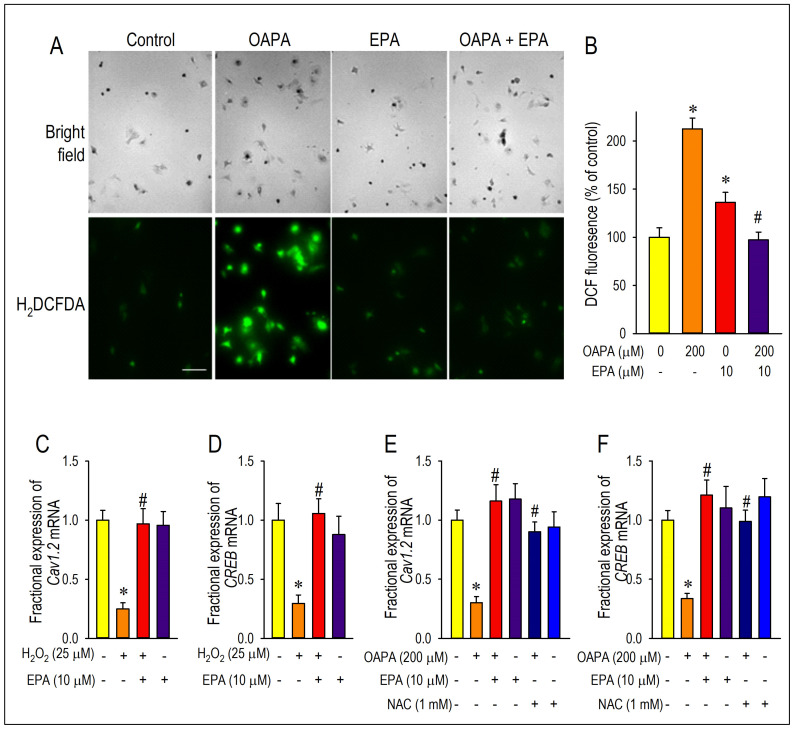
Demonstration of actions of ROS for the expression of Cav1.2 and CREB. Cardiomyocytes were incubated with OAPA (200 μM) in the presence or absence of EPA (10 μM) for 24 h and stained with CMH_2_DCFDA. Representative images (**A**) and quantitative results are shown (**B**). Fluorescence intensity of non-treated myocytes (OAPA (-), EPA (-)) was set as 100%., and data are expressed as mean ± SE. Scale bar = 50 μm. (**C**,**D**) Regulation of *Cav1.2* and *CREB* mRNA expression by oxidative stresses using H_2_O_2_ (25 μM) applied for 24 h. (**E**,**F**) Effects of ROS scavenger on *Cav1.2* and *CREB* mRNA expressions. Cardiomyocytes were exposed to OAPA (200 μM) for 24 h in the presence or absence of EPA (10 μM) with/without an ROS scavenger NAC (1 mM). Data are expressed as mean ± SE (n = 6). * *p* < 0.05, vs. non-treated cardiomyocytes (OAPA (-), EPA (-)) (**B**), (H_2_O_2_ (-), EPA (-)) (**C**,**D**) or (OAPA (-), EPA (-), NAC (-)) (**E**,**F**). # *p* < 0.05, vs. cardiomyocytes (OAPA (+), EPA (-)) (**B**), (H_2_O_2_ (+), EPA (-)) (**C**,**D**) or (OAPA (+), EPA (-), NAC (-)) (**E**,**F**).

Although the downregulation of *Cav1.2* and *CREB* mRNA expression caused by OAPA and the rescue by EPA were confirmed, their signal pathways are largely unknown. To clarify the intracellular signal pathway responsible for the action of EPA, the functional role of the EPA receptor FFAR4 was examined. To our knowledge, the function of FFAR4 is poorly known in cardiac tissue and/or cardiomyocytes. We investigated whether FFAR4 could be detected in the adult mouse heart and neonatal mouse ventricular cardiomyocytes by using real-time PCR analysis. Figure 5A clearly demonstrates that FFAR4 is detectably expressed in adult and neonatal hearts. Notably, there was an approximately 25-fold difference in *FFAR4* mRNA expression between the atrium and the ventricle in the adult mouse heart; surprisingly, *FFAR4* was highly expressed in the atrium. Based on the result that *FFAR4* was appreciably expressed in neonatal ventricular cardiomyocytes, FFAR4-mediated signal pathways to regulate Cav1.2 and CREB were examined by use of neonatal mouse ventricular cardiomyocytes; an FFAR4 antagonist AH7614 and an FFAR4 agonist TUG-891 were applied [36]. It is worthy of note that the actions of EPA to rescue *Cav1.2* and *CREB* were blocked by an FFRA4 antagonist AH-7614 (Figure 5B,C). Even more, the reduction in *Cav1.2* and *CREB* mRNA caused by OAPA was rescued by an FFAR4 agonist TUG-891 mimicking the effect of EPA (Figure 6B,C). These results firmly suggest the mechanism of FFAR4-mediated L-type Ca^2+^ channel regulation pathway led by EPA in cardiomyocytes.

### 2.4. Actions of ROS for Cav1.2 Expression

To investigate the effect of OAPA treatment on the level of oxidative stress, the amount of reactive oxygen species (ROS) accumulation was measured in cardiomyocytes. As shown in Figure 6A,B, the level of ROS was relatively low in cardiomyocytes exposed to the normal culture medium. On the other hand, OAPA treatment caused ROS accumulation in cardiomyocytes, which was rescued by EPA co-treatment. Intriguingly, EPA alone increased the ROS accumulation to a certain degree, although we did not examine the action any further in this study. Based on these results, it is suggested that EPA may act as a scavenger to remove ROS in cardiomyocytes. Roles of ROS for the modulation of Cav1.2 and CREB expression were further examined by use of hydrogen peroxide (H_2_O_2_) which induces oxidative stress in cardiomyocytes (Figure 6C,D). When cardiomyocytes were loaded with H_2_O_2_ for 24 h, the expression of *Cav1.2* and *CREB* mRNA was markedly suppressed. However, the simultaneous administration of EPA nearly completely rescued the reduction in *Cav1.2* and *CREB* mRNA. The ROS scavenging effects on OAPA insults for expression of *Cav1.2* and *CREB* mRNA were examined by use of an ROS scavenger N-acetylcysteine (NAC) in comparison with EPA. NAC distinctly rescued expression of *Cav1.2* and *CREB* mRNA, suggesting that OAPA downregulates Cav1.2 and CREB via the mechanism associated with ROS accumulation. Taken together, it is concluded that OAPA-induced ROS accumulation modulates Cav1.2-L-type Ca^2+^ channel transcription through an FFAR4-independent pathway.

## 3. Discussion

The major findings of this study were that (1) the long-term application of OAPA decreased *Cav1.2* mRNA/protein, *I*_Ca.L_ and the spontaneous beating of cardiomyocyte, (2) EPA application reversed the decline of Cav1.2 channel caused by OAPA, (3) an FFAR4 agonist TUG-891 reversed expression of *Cav1.2* and *CREB* mRNA caused by OAPA, (4) an FFAR4 antagonist AH-7614 abolished the effects of EPA on *Cav1.2* and *CREB* mRNA caused by OAPA, (5) OAPA increased ROS production, while the action was eliminated by EPA, (6) an ROS generator H_2_O_2_ decreased the expression of *Cav1.2* and *CREB*, which was prevented EPA, and (7) suppressions of *Cav1.2* and *CREB* mRNA by OAPA was prevented by an ROS scavenger NAC.

**Figure 7:**
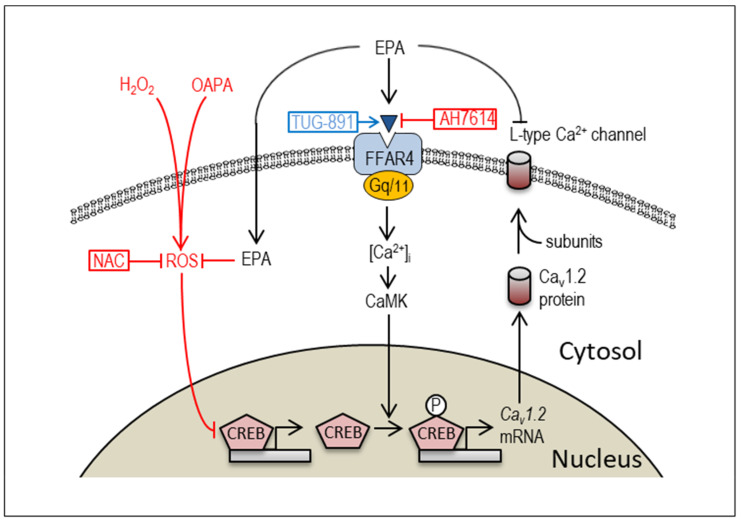
The proposed molecular mechanism of EPA and OAPA on the Cav1.2-L-type Ca^2+^ channel expression. EPA modulates Cav1.2 expression via FFAR4-depenent and -independent signal pathways apart from the acute inhibitory action on the L-type Ca^2+^ channel [26]. Postulated actions of an ROS scavenger NAC, a selective FFAR4 antagonist AH7614, and an FFAR4 agonist TUG-891 on CREB-associated pathways are also shown.

Intracellular Ca^2+^ homeostasis is a critical determinant of cardiac function, and the levels of intracellular Ca^2+^ are cooperatively regulated by the sarcolemmal Ca^2+^-ATPase, several types of the Ca^2+^ channels, the sodium–calcium exchanger, and other regulatory proteins. At the same time, intracellular Ca^2+^ signaling plays an essential role in cardiac gene expression and cardiogenesis [37,38]. In this context, it is not surprising that EPA modifies the expression of Cav1.2 through the intracellular Ca^2+^ regulatory pathway (Figure 7). We believe that the present study reveals the novel mechanism via which EPA administration rescues the downregulation of voltage-gated L-type Ca^2+^ channels caused by excessive amounts of saturated fatty acids and/or ROS in cardiomyocytes. In recent years, although little is known about the intracellular molecular mechanisms by which EPA exerts cardioprotective effects in cardiomyocytes, there has been increasing interest in FFAR4, a selective receptor for EPA [33]. Because EPA-induced FFAR4 activation is known to signal through modulation of Gq/11 proteins to stimulate CaMK activity in various cell types [33,36], and because CREB-dependent regulation of Cav1.2 channel transcription has been reported in cardiomyocytes [35], it is proposed that EPA regulates Cav1.2 channel expression possibly through the FFAR4-CaMK-CREB signaling pathway (Figure 7). Of note, FFAR4-independent signal pathway of EPA for the regulation of Cav1.2 expression is also proposed in this study. Since EPA has many double bonds and long-chain carbons, the incorporation of EPA into the plasma lipids within the plasma membrane can alter its properties and influence the function of various membrane proteins including ion channels and receptors [27]. Furthermore, the intracellular concentration of EPA can easily be upregulated following extracellular EPA treatment [39]. Taken together, the ROS-scavenging effects of EPA to upregulate CREB and Cav1.2 could be attributed to its FFAR4-independent pathway at the same time. Interestingly, ROS generation by OAPA and ROS scavenging effect of PUFA are well recognized in various types of cells including cardiomyocytes [40,41]; PUFAs are known to activate antioxidant enzymes such as catalase and superoxide dismutase [41]. Although the cardioprotection effect of PUFAs are documented by their antioxidant mechanism [41], the mechanism of their regulatory actions on ion channel transcription is largely unknown, which obviously needs to be further elucidated.

Several studies have revealed an association between omega-3 PUFA intake and a lower risk of cardiovascular events including arrhythmias. More specifically, omega-3 PUFA is known to modify cardiac excitability by regulating ion channel functions in cardiomyocytes [42,43]. EPA is believed to prevent atrial and ventricular arrhythmias in animal experiments by inhibiting voltage-gated ion channels such as the Na^+^ channel [43,44], the Ca^2+^ channels [45], and some types of the K^+^ channels [46]. These findings suggest that the antiarrhythmic actions of EPA are mediated by direct interaction with membrane ion channels. On the contrary, long-term beneficial actions of PUFAs were to limit atrial remodeling that predispose patients to develop atrial fibrillation [47]. These observations suggest that PUFAs may act as antiarrhythmic nutrients to modify cardiac excitability when applied for long-term periods as well. Several independent studies have reported the effects of PUFAs to modulate expressions of ion channels in the heart; EPA suppressed expression levels of K^+^ channels and their related genes, *Kir6.2*, *Kcna5*, *Kcnd2*, *KChIP2* [48,49], and Na^+^ channel mRNA [50]. Interestingly, in partially agreement with our findings, Xu at al. recently demonstrated that long-term application of fish oil upregulated the expression of Cav1.2 channel protein in the rabbit heart [46], which is in the sharp contrast to the previous studies reporting an antagonistic action of EPA to the voltage-gated Ca^2+^ channel [51]. A potentiation of the Ca^2+^ channel by long-term application of EPA could be accordingly considered as a compensatory action that maintains intracellular Ca^2+^ concentration reduction caused by the short-term effect. However, the long-term effect of EPA on the Ca^2+^ channel may be more complicated than the above postulation. It is known that ingested EPA is present in the blood in the form of phospholipids. PUFAs bind to and are incorporated into phospholipids in cell membranes because they are structurally unsaturated. EPA may affect membrane fluidity, lipid microdomain formation, and trans-membrane signaling. Consistently, an animal study demonstrated a significant increase in EPA and DHA concentrations in the ventricular myocardium of mouse supporting the notion that EPA acts, at least in part, directly on cardiomyocytes to maintain the electrical properties of ion channels present on the cell membrane [46]. Although this study did not assess the mechanism further, a high concentration of EPA can reduce the expression of the Ca^2+^ channel by itself (Figure 1C). Considering that the human serum concentration of EPA approximately ranges from 14 μM to 100 μM [52], it is assumed that the long-term action of EPA on the Ca^2+^ channel ex pression could be affected by various factors including concentrations of EPA and co-existence of other saturated/unsaturated fatty acids, FFAR4 density on the plasma membrane, endogenous intensity of CaMK signals, and the magnitude of CREB-dependent transcription in cells. Although several publications describe that omega-3 fatty acids prevent ventricular arrhythmias [53], a much larger number of studies document that EPA prevents atrial fibrillation in animal studies and clinical observation [53,54]. It is crucial to note that the expression level of FFAR4 in the atrium was 25 times larger than that in the ventricle (Figure 5A). Although electrophysiological actions of EPA on cardiomyocytes appear not only via FFAR4, a dense distribution of FFAR4 in the atrium suggests that actions of EPA on the Ca^2+^ channel are highly expected in the atrium. A negative chronotropic effect of EPA [55] may also be associated with the FFAR4 expression distinction in the heart. Relatively high concentrations of EPA by itself reduced expression of *Cav1.2 mRNA* (Figure 1C), in spite of the fact that EPA rescued the reduction in them caused by OAPA. Taken together, it is suggested that EPA is able to decrease automaticity or responsiveness of the sinus node. Because the sinus node is located within the atrial tissue and is functionally connected to the atrial cardiomyocytes, the impact of FFAR4 activation in the atrium could influence pacemaker rhythm accordingly. In humans, the expression of FFAR4 decreases in cardiomyocytes with heart failure [33]. Thus, it is likely that expression of FFAR4 in the atrium may change in the pathological conditions such as in atrial fibrillation, suggesting the importance of FFAR4 density in the evaluation of EPA action on the pathological condition of the heart. 

Limitations of this study include uncertainty about whether these results are directly applicable to humans with dyslipidemia. Results in this study were obtained from animal experiments with isolated mouse/rat cardiomyocytes and in vitro analysis that used real-time PCR and patch clamp analysis but was not applied to human body. Although the impact of an FFR4 agonist TUG-891 on the expression of Cav1.2 and CREB was robust, it is uncertain whether the FFAR4-dependent effect of EPA is more dominant than the FFAR4-independent effect to rescue the L-type Ca2+ channel, which needs further studies. Also, the underlying mechanisms that connect FFAR4 and Cav1.2 remain largely unclear and require further investigation.

## 4. Materials and Methods

### 4.1. Chemicals

Reagents were obtained from Sigma Aldrich (St. Louis, MO, USA) or WAKO (Osaka, Japan) unless otherwise indicated. Triton X-100 was purchased from MP Biomedicals (Aurora, OH, USA). Fetal bovine serum was obtained from Biosera (Biosera, Nuaillé, Chile). Collagenase type IV was purchased from Worthington (Lakewood, NJ, USA). H_2_DCF-DA (2′,7′-dichlorodihydrofluorescein diacetate), ProLong Diamond Antifade Mountant with 4′,6-diamidino-2-phenylindole dihydrochloride (DAPI) were from Molecular Probes (Eugene, OR, USA). Anti-CREB antibody (1:1000, Cell Signaling, Beverly, MA, USA), phosphospecific antibody against anti-pCREB (Ser 133), and Alexa Fluor 488 and 594-conjugated secondary antibodies were from Cell Signaling (Danvers, MA, USA). All reagents from commercial sources were of analytical grade.

### 4.2. Isolation of Neonatal Mouse Cardiomyocytes

C57BL/6 mouse and Wistar rats (Japan SLC, Inc., Shizuoka, Japan) were provided with food and water ad libitum and the room temperature was maintained at 25 ± 1 °C in a 12 h light/12 h dark cycle. Neonatal mouse or rat cardiomyocytes were enzymatically isolated and cultured as previously described [56,57]. The cardiomyocytes were maintained at 37 °C under 5% CO_2_ in Dulbecco’s modified Eagle’s medium (DMEM) supplemented with 10% fetal bovine serum for 24 h. After 24 h of culture, >70% of the cells adhered to the substrates and started to exhibit spontaneous beating. For electrophysiological experiments, we used isolated neonatal rat cardiomyocytes.

### 4.3. Preparation of OAPA and Cell Culture

Oleic acid (OA) was solubilized in anhydrous methanol, and then 100 mM stock solution was prepared. Palmitic acid (PA) bovine serum albumin (BSA) conjugate was prepared through soaping PA with 0.5 N sodium hydroxide (NaOH) and mixing with BSA. Briefly, 100 mM stock solution of PA in 0.5 N NaOH was incubated at 70 °C for 30 min. Then, we prepared a mixture in OA at a concentration of 500 μM and PA at a concentration of 250 μM. Both fatty acids were complexed to BSA in a ratio of 2:1 (OA:PA) and total concentration of 100–500 μM. Because in vitro studies have shown that the PA at physiological concentrations exhibits a dose-dependent cytotoxic effect associated with ROS production and apoptosis or necrosis in neonatal cardiomyocytes [9], we tried to examine a combination of OA with PA, which is more suitable for simulation of simple electrical changes than PA alone. Medium supplementation with OA (500 μM)/PA (250 μM) mixture (OAPA) in the presence or absence of EPA (10 μM) for 24 h. To investigate the effect of removing accumulated ROS, cardiomyocytes were exposed to an OAPA for 24 h in the presence or absence of 1 mM NAC, an ROS scavenger.

### 4.4. Measurement of Intracellular ROS Accumulation

Intracellular ROS accumulation was detected using H_2_DCFDA, whose green fluorescence signal is increased by its oxidation by ROS. Cells were incubated with 2 μM H_2_DCFDA for 50 min at 37 °C and rinsed with HEPES buffer twice before observation. Images were acquired and digitized on a BIOLEVO BZ-9000 epifluorescence microscope (Keyence, Osaka, Japan), and analyzed at 200× magnification using the associated software (Keyence).

### 4.5. Electrophysiological Measurements

Whole-cell voltage clamp experiments were performed as described previously [58]. L-type Ca^2+^ channel current (*I*_Ca.L_) was recorded from a holding potential (V_H_) of −50 mV followed by various test potentials. *I*_Ca.L_ density was obtained by normalizing *I*_Ca.L_ to the cell capacitance. All experiments were conducted at 37 °C. For measuring *I*_Ca.L_, the bath solution was composed by Na^+^- and K^+^-free solution contained (mM): Tetraethy-lammonium chloride (TEA-Cl) 120, CsCl 6, 4-aminopyridine (4-AP) 5, MgCl_2_ 0.5, 4,4P-diisothiocyanostilbene-2,2P-disulfonic acid (DIDS) 0.1, HEPES 10, CaCl_2_ 1.8, and glucose 10 (pH of 7.4 adjusted with TEA-OH). The pipette solution contained (mM): CsCl 130, Mg-ATP 2, EGTA 5, and HEPES 10 (pH of 7.2 adjusted with 1 M CsOH).

### 4.6. Quantitative Real-Time PCR

Total RNA was extracted from rat neonatal ventricular cardiomyocytes using TRIzol (Invitrogen, Carlsbad, CA, USA) 24 h after the treatment with agents described above. The single-stranded cDNA was synthesized from 1 µg of total RNA using Transcriptor First Strand cDNA Synthesis Kit (Roche Molecular System Inc., Alameda, CA, USA). Real-time PCR was performed on Light Cycler (Roche) using the FastStart DNA Master SYBR Green I (Roche) as a detection reagent. Forward and reverse primer sequences, respectively, for mouse L-type Ca^2+^ channel isoforms and transcription factor were designed from their sequence in the GeneBank database as follows (accession numbers are indicated in parentheses): CACNA1C (NM_001255999), forward 5′-ACATCTTCGTGGGTTTCGTC-3′, reverse 5′-TGTTGAGCAGGATGAGAACG-3′; CACNA1D (NM_028981), 5′-CTTTTGGAGCCTTCTTGCAC-3′, reverse 5′-CTGGACTGAATCCCAAAGGA-3′; FFAR4 (NM_181748), forward 5′-GCCCAACCGCATAGGAGAAA-3′, reverse 5′-GTCTTGTTGGGACACTCGGA-3′; CREB (NM_133828), forward 5′-TGGAGTTGTTATGGCGTCCT-3′, and reverse 5′-CGACATTCTCTTGCTGCCTC-3′. Glyceraldehydes-3-phosphate dehydrogenase (GAPDH; GU214026) mRNA was used as an internal control. Data were calculated by 2^−∆∆CT^ and presented as fold change in transcripts for Cav1.2 genes in myocytes and normalized to GAPDH (defined as 1.0 fold).

### 4.7. Western Blot Analysis

Cultured neonatal mouse cardiomyocytes were treated with OAPA in the presence or absence of EPA for 24 h in DMEM. After the treatments, cardiomyocytes were washed twice with ice cold PBS and harvested using cell scraper, and then lysed in RIPA buffer containing protease inhibitor mixture and phosphatase inhibitors on ice for 1 h. The extracted protein concentration was determined by the BCA protein assay kit (Pierce). Samples containing 40 μg were denatured at 95 °C for 5 min in loading buffer [Tris-HCl (pH 6.8) 250 mM, 4% SDS, 1% β-mercaptoethanol, 1% bromophenol blue, and 20% glycerol], separated by SDS polyacrylamide gel electrophoresis using 10% polyacrylamide gel, and then transferred from the gel to a PVDF membrane (Hybond-P; GE Healthcare Bio-Sciences, Piscataway, NJ, USA). To prevent nonspecific binding, the blotted membranes were blocked with 5% skim milk in tris-buffered saline (TBS) with 0.1% Tween 20 (TBST) for 1 h at room temperature and then probed overnight at 4 °C with an anti-CREB antibody (1:1000, Cell Signaling, Beverly, MA, USA), phosphospecific antibody against anti-pCREB (Ser 133) (1:1000, Cell Signaling), and Cav1.2 (1:200, Alomone Labs, Jerusalem, Israel). The blot was visualized with anti-rabbit IgG horseradish peroxidaseconjugated secondary antibodies (1:2000, American Qualex, San Clemente, CA, USA) and an ECL prime Western Blotting Detection System (GE Healthcare Bio-Sciences). GAPDH antibody (1:1000, Proteintech, Wuhan, China) was used as loading control. The relative target protein levels were quantified by densitometry normalized to GAPDH on the same membrane. Band density was measured using Image J 1.51 software (National Institute of Health, Bethesda, MD, USA).

### 4.8. Immunocytochemistry

The details of the experiments protocol were performed as described previously [56]. Primary antibodies against Phospho-CREB (1:200, Cell Signaling, Beverly, MA, USA) and Cav1.2 (1:200, Alomone Labs Ltd., Jerusalem, Israel) were applied following incubation with the appropriate fluorescence-labeled secondary antibodies (Cell Signaling) for 1 h at room temperature. After several washes, the samples were air-dried, mounted with a drop of ProLong Diamond Antifade Mountant with DAPI (Molecular Probes) and subjected to microscopy. Images were obtained with a confocal microscope system (A1R, Nikon, Tokyo, Japan) equipped with a PlanFluor 60× objective lens and excitation lasers (488 and 561 nm, Melles Griot, New York, NY, USA). Images were saved in TIFF format and analyzed by ImageJ software (Wayne Rasband, National Institutes of Health).

### 4.9. Statistical Analysis

Statistical analysis was conducted using SigmaPlot 14.0 (SigmaPlot version 14.0-Systat Software, Inc., London, UK). All data are expressed as mean ± SE. The significance of differences was determined by one-way ANOVA followed by Tukey’s test. Values of *p* < 0.05 were considered statistically significant.

## Data Availability

The data in this study are available from the corresponding authors on reasonable request.
